# The Cost-Effectiveness of Avatrombopag Versus Eltrombopag and Romiplostim in the Treatment of Patients with Immune Thrombocytopenia in the UK

**DOI:** 10.3390/jmahp13020011

**Published:** 2025-03-24

**Authors:** Nichola Cooper, Sebastian Guterres, Michał Pochopień, Koo Wilson, Sam James, Mondher Toumi, Anna Tytuła, Carly Rich, Daniel Eriksson

**Affiliations:** 1 Faculty of Medicine, Department of Immunology and Inflammation, Imperial College London, London SW7 2AZ, UK; 2 Swedish Orphan Biovitrum Ltd., Cambridge CB21 6AD, UK; sebastian.guterres@sobi.com (S.G.); sam.james@sobi.com (S.J.); 3 Assignity, 30-415 Kraków, Poland; mpo@assignity.com; 4 Swedish Orphan Biovitrum AB, SE-112 76 Stockholm, Sweden; koo.wilson@sobi.com (K.W.); carly.rich@sobi.com (C.R.); daniel.eriksson@sobi.com (D.E.); 5 Department of Public Health, Aix-Marseille University, 13005 Marseille, France; mto@inovintell.com; 6 Health Economics and Outcomes Research Department, Putnam PHMR, 30-701 Kraków, Poland; anna.tytula@putassoc.com

**Keywords:** avatrombopag, cost-effectiveness, eltrombopag, immune thrombocytopenia, incremental cost-effectiveness ratio, romiplostim, thrombopoietin receptor agonists

## Abstract

Background: Thrombopoietin receptor agonists—romiplostim, eltrombopag and avatrombopag—are commonly used as second-line treatments for immune thrombocytopenia (ITP). Methods: A Markov model was developed to estimate the cost effectiveness of the three TPO-RAs in adults with insufficient response to previous treatment from the perspective of the UK National Health Service (NHS). The model considered the effects of bleeding events, concomitant ITP medications, rescue therapies and treatment related adverse events over a lifetime horizon. Model inputs for effectiveness were based on a network meta-analysis and other published literature on ITP management. Other model inputs included costs (e.g., drug acquisition and administration) and healthcare resource utilisation. Results: Avatrombopag was associated with higher quality-adjusted life-years (QALYs) (10.979) than romiplostim (10.628) and eltrombopag (10.085), producing incremental QALYs of −0.351 and −0.894, respectively. Avatrombopag was associated with lower total costs (GBP £319,334) compared with romiplostim (GBP 406,361 [cost saving of GBP 87,027]) and higher total costs compared with eltrombopag (GBP 313,987 [incremental cost of GBP 5347]). Avatrombopag therefore dominated romiplostim (more effective and less expensive) and was cost-effective versus eltrombopag (incremental cost-effectiveness ratio of GBP 5982 per QALY). Conclusions: Avatrombopag is a cost-effective treatment compared with romiplostim and eltrombopag for the second-line treatment of adults with ITP from the perspective of the UK NHS.

## 1. Introduction

Immune thrombocytopenia (ITP) is an autoimmune disease characterised by reduced platelets levels (<100 × 10^9^/L) due to immune-mediated destruction and diminished platelet production in the bone marrow, and an increased risk of bleeding [1,2,3,4]. It is classified as primary or secondary to other autoimmune diseases, viral infections, specific drugs or vaccinations. Primary ITP accounts for approximately 80% of adult ITP cases [1].

Clinically, ITP is characterised by mucocutaneous bleeding [5], with symptoms ranging from mild bruising to serious, potentially fatal, haemorrhage [6]. Significant bleeding is rare in those with a platelet count > 30 × 10^9^/L, but there is substantial variability in bleeding risk among patients [6]. Treatment is initiated in patients with bleeding diathesis and possibly also in asymptomatic cases where the platelet count is <20–30 × 10^9^/L; treatment aims to improve platelet count and achieve haemostasis [2,7]. First-line treatment options for the management of primary ITP include corticosteroids and intravenous immunoglobulin (IVIg) [7]. Corticosteroids remain the standard initial treatment for newly diagnosed patients but are generally used for a limited time only as their side effects outweigh their benefits over prolonged periods [7]. Most adults (70–80%) relapse with cessation of steroid treatment and as a result, subsequent therapy is indicated [7,8,9].

Second-line options for ITP include thrombopoietin receptor agonists (TPO-RAs) and rituximab (unlicensed for ITP) [7], while fostamatinib is an option in the United Kingdom (UK) for patients with refractory ITP previously treated with a TPO-RA, or if a TPO-RA is unsuitable [10]. Other unlicensed treatments include immunosuppressants such as azathioprine and mycophenolate mofetil [7]. TPO-RAs work by stimulating the proliferation and differentiation of megakaryocytes from bone marrow progenitor cells, providing increased production of platelets [11]. The response to continued TPO-RA treatment persists over many years and often allows for other ITP therapies to be reduced or discontinued [7]. Avatrombopag is the most recent TPO-RA to be licenced in Europe and the United States of America (USA) and represents an important addition to the therapeutic armamentarium for ITP. Three TPO-RAs, romiplostim, eltrombopag and avatrombopag, have been assessed and recommended as second-line options for primary ITP by the National Institute for Health and Care Excellence (NICE).

No head-to-head studies comparing romiplostim, eltrombopag and avatrombopag are published. To address this, several systematic literature reviews (SLRs) and network meta-analyses (NMAs) were conducted to assess the efficacy and/or safety of TPO-RAs as second-line treatments in patients with ITP [12,13,14,15,16,17,18,19]. Overall, these analyses reported that TPO-RAs were more effective and provide similar tolerability/safety to placebo. The NMAs by Deng et al. and Li et al. reported that avatrombopag provided a significantly better platelet response rate than eltrombopag and romiplostim [16,17], while Liu et al. reported a significantly better platelet response with avatrombopag versus eltrombopag [12]. Similarly, the NMA by Wojciechowski et al. reported that avatrombopag was associated with a statistically significant lower incidence of any bleeding events versus eltrombopag and romiplostim [13]. Also, no between-treatment differences for the other endpoints, including need for rescue treatment and adverse events (AEs), were observed [13]. The other two studies that reported data for individual treatments (Arai et al. and Yang et al.) found no significant differences in response rates or AEs between the TPO-RAs [14,19]. In addition to efficacy and safety, other factors to consider when making decisions on the management of ITP with TPO-RAs include patient comorbidities, patient preferences and drug costs [2,20].

The aim of the present study was to develop an evidence-based tool to estimate the cost-effectiveness of avatrombopag compared with eltrombopag and romiplostim for the treatment of adults with ITP.

## 2. Materials and Methods

### 2.1. Model Overview

A cohort Markov model was developed to assess the cost-effectiveness of avatrombopag compared with eltrombopag and romiplostim. Its structure was based on previous TPO-RA submissions to NICE [21,22] and other published cost-effectiveness models for TPO-RAs [23,24]. Data inputs for the model were derived from the NMA conducted by Wojciechowski et al. [13] (in the absence of head-to-head data) and other clinical and health economic sources.

The model considered a hypothetical cohort of adults with ITP with an insufficient response to previous treatment, the licenced indication for the TPO-RAs. A Markov state-transition model with embedded decision tree was developed, with a 4-week cycle and a lifetime horizon. The analysis was conducted from the perspective of the UK National Health Service (NHS). As per the NICE reference case [25], a discount rate of 3.5% was applied for health outcomes and costs (applicable to all three TPO-RAs). Sensitivity analyses were conducted to assess the validity of the model.

### 2.2. Model Description

**Figure 1:**
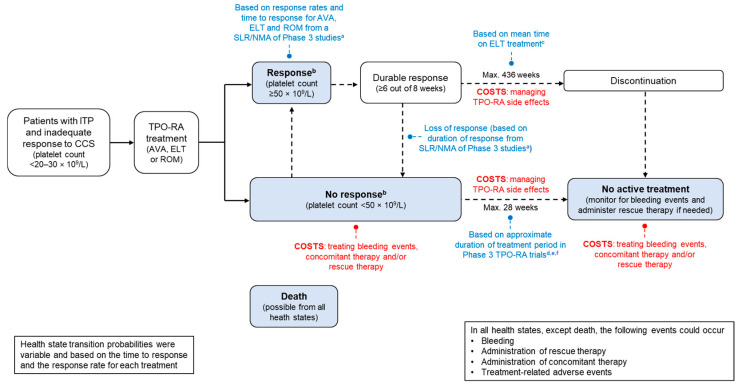
Model structure. ^a^ Wojciechowski P et al. [13]. ^b^ Utilities for ‘no bleed’ state were 0.801 (for patients with platelet levels ≥ 50 × 10^9^/L) and 0.760 (patients with platelet levels < 50 × 10^9^/L). ^c^ Lee D et al. [24]. ^d^ Jurczak W et al. [26]. ^e^ Cheng G et al. [27]. ^f^ Kuter DJ et al. [28]. AVA: avatrombopag; CCS: corticosteroids; ELT: eltrombopag; ITP: immune thrombocytopenia; NMA: network meta-analysis; ROM: romiplostim; SLR: systematic literature review; TPO-RA: thrombopoietin receptor agonist.

The model simulates patients with chronic ITP receiving avatrombopag, eltrombopag or romiplostim. An overview of the model, including inputs and outputs, is provided in Figure 1, and each element of the model is described in more detail below.

#### 2.2.1. Treatment Pathway

At model entry, patients with ITP not responding adequately to corticosteroids (platelet count < 20–30 × 10^9^/L) were assigned to active treatment with avatrombopag, eltrombopag or romiplostim. Patients were defined by treatment response (platelet count ≥ 50 × 10^9^/L) or non-response (platelet count < 50 × 10^9^/L) to the TPO-RAs, consistent with the end points defined in clinical trials.

#### 2.2.2. Health States

The model included four possible health states: (1) ‘Response’; (2) ‘No response’; (3) ‘No active treatment’; and (4) ‘Death’ (Figure 1). Patients could change health states every 4-week cycle based on the assumption that follow-up of all patients would undergo a haematologist consultation, blood test and biochemistry each month. The probabilities of transitioning across health states were variable and based on the time to response and the response rate for each treatment.

Patients in the ‘Response’ health state responded to active treatment and either remained in this state (if they had a durable response) until treatment discontinuation (an assumed average treatment duration of 109 cycles [436 weeks]) or until loss of response. Those with loss of response entered the ‘No active treatment’ health state. Patients in the ‘No response’ health state had the potential to achieve a response to active treatment, but if they did not respond within 28 weeks (the approximate duration of the treatment period in the Phase 3 TPO-RA trials), they transitioned to the ‘No active treatment’ state. During the ‘No active treatment’ state, concomitant and rescue therapies were permitted.

Bleeding events, administration of concomitant ITP medication, administration of rescue therapy and/or treatment-related AEs (TRAEs) could occur in all health states except death. It was assumed that achieving a platelet response would lead to a reduction in the risk of bleeds and the need for rescue and/or concomitant therapies.

### 2.3. Model Inputs

The key model base-case input values and their sources for the three TPO-RAs are summarised in Table 1 [7,13,21,22,24,26,27,28,29,30,31,32,33,34,35] and the corresponding information related to concomitant and rescue therapies are summarised in Table S1 [21,26,36,37,38].

#### 2.3.1. Efficacy

The efficacy of each active treatment in the model was characterised by time to response (time taken to achieve platelet levels ≥ 50 × 10^9^/L), response rate (proportion of patients achieving a response) and duration of response (time period when platelet levels remained ≥ 50 × 10^9^/L) (Table 1). The probabilities of durable platelet response (defined as platelet level of ≥50 × 10^9^/L for at least 6 of the last 8 weeks of treatment) for avatrombopag, eltrombopag and romiplostim were derived from a previously published NMA, a well-established method for comparing treatments in the absence of head-to-head trials [39], which was conducted using a Bayesian framework [13] (Table S2). Relevant studies for the NMA were identified by an SLR and the analyses were conducted in accordance with the Preferred Reporting Items for Systematic Review and Meta-Analyses (PRISMA) guidelines [40]. As no durable responses were reported in the placebo arms of the avatrombopag and romiplostim (splenectomised) trials, a zero-correction factor was applied, based on the proportion of participants in each trial arm (as advocated by Sweeting et al. in the case of unbalanced treatment arms [41]).

**Table 1:** Data sources and values for key model inputs.

Patient Demography and Clinical Outcome Parameters.		
Input	Value	Source
Patient demography	Mean age: 44.6 years Sex, male: 36.7% Mean body weight: 82.97 kg Mean body area: 1.94 m^2^	Based on data from avatrombopag Phase 3 trial [26]
Durable response rates	Avatrombopag: 11/32 (34.4%) Placebo: 0/17 (0%) Eltrombopag: 57/95 (60.0%) Placebo: 4/39 (10.3%) Romiplostim (splenectomised patients): 16/42 (38.1%) Placebo (splenectomised patients): 0/21 (0%) Romiplostim (non-splenectomised patients): 25/41 (61.0%) Placebo (non-splenectomised patients): 1/21 (4.8%)	Data from individual clinical trials [26,27,28], as summarised by Wojciechowski et al., 2021 [13]
Relative probability of durable response (≥50 × 10^9^/L)	Base case (Bayesian framework) Avatrombopag: 73% (95% CI, 9–100%) Eltrombopag: ^a^ 27% (12–59%) Romiplostim: ^a^ 55% (19–95%) Scenario analysis (frequentist framework) Avatrombopag: 42% (NC) Eltrombopag: 22% (NC) Romiplostim: 47% (NC)	Calculation based on data from Wojciechowski et al., 2021 [13] Calculation (based on an NMA, conducted using a frequentist framework)
Time to response (≥50 × 10^9^/L)	TPO-RAs: 24 weeks (6 cycles) Concomitant and rescue therapies: see Table S1	Based on time to durable response from individual trials [26,27,28]
Duration of response	TPO-RAs: 109 cycles Concomitant and rescue therapies: see Table S1	Mean time on eltrombopag treatment, based on fitting log-normal curves to eltrombopag data published in Lee 2013 [24]
Proportion of patients with bleeding type (per cycle)	Platelets ≥ 50 × 10^9^/L Minor bleed: ^b^ 10.0% Outpatient bleed: 7.1% Inpatient bleed: 0% Platelets < 50 × 10^9^/L Minor bleed: ^b^ 17.1% Outpatient bleed: 45.5% Inpatient bleed: 4.3%	Eltrombopag NICE submission [22] and patient-level data from avatrombopag Phase 3 trial [26]
Distribution of types of bleeds requiring hospitalisation ^c^	Platelets ≥ 50 × 10^9^/L Intracranial haemorrhage: 0% Gastrointestinal bleed: 29% Other inpatient bleed: 71% Platelets < 50 × 10^9^/L Intracranial haemorrhage: 19% Gastrointestinal bleed: 19% Other inpatient bleed: 63%	Based on eltrombopag NICE submission [22]
**Costs.**		
**Input**	**Value**	**Source**
Bleeding costs	Increased NHS tariff ^d^ (base case) Outpatient: GBP 494 Intracranial: GBP 7044 Gastrointestinal: GBP 5503 Other inpatient: GBP 3485 Standard NHS tariff (scenario analysis) Outpatient: GBP 460 Intracranial: GBP 4691 Gastrointestinal: GBP 3092 Other inpatient: GBP 2891 Data from qualitative study (scenario analysis) Outpatient: GBP 3134 Intracranial: GBP 25,699 Gastrointestinal: GBP 14,325 Other inpatient: GBP 14,325	Increased NHS tariff Highest listed NHS tariff costs for each type of bleed corresponding to those with the highest complication and comorbidity score (i.e., the most severe bleeds) [29]; see Supplementary File S1 ^e^ for further information Standard NHS tariff Weighted average of NHS unit (or tariff) costs associated with different complication and comorbidity scores from NHS reference costs (i.e., accounts for all severities of bleed) [29]; see Supplementary File S1 ^e^ for further information Qualitative study Based on data from Pogna et al., 2021 [30]; see Supplementary File S1 ^e^ for further information
Drug acquisition costs (list price)	Avatrombopag: GBP 1920 (30 × 20 mg) Eltrombopag: GBP 1540 (28 × 50 mg) Romiplostim: GBP 241 (0.125 mg) Concomitant and rescue therapies: see Table S1	Avatrombopag: Sobi Eltrombopag and romiplostim: NICE British National Formulary 2021 [42]
Dosing—TPO-RA	Avatrombopag: 20 mg/day Eltrombopag: 50 mg/day Romiplostim: 0.004 mg/kg per week ^f^	Avatrombopag SmPC [43] Eltrombopag SmPC [44] Kuter et al., 2010 [32]
Drug administration costs	Romiplostim ^g^: GBP 241.06 per infusion Concomitant and rescue therapies: see Table S1	NHS reference costs 2018/2019 (weighted average cost) [38]
Follow-up costs ^h^	Haematologist consultation: GBP 173.39 Blood test: GBP 2.79 Biochemistry: GBP 1.10	NHS reference costs 2019/2020 (HRG codes: 303 Clinical Haematology, consultant led; DAPS05 Haematology; DAPS04 Clinical Biochemistry) [31]
**Utilities.**		
**Input**	**Value**	**Source**
Utilities	Platelets ≥ 50 × 10^9^/L No bleed: 0.801 Outpatient bleed: 0.625 Inpatient bleed: intracranial haemorrhage, 0.038; gastrointestinal bleed, 0.45; other inpatient bleed, 0.45 Platelets < 50 × 10^9^/L No bleed: 0.760 Outpatient bleed: 0.584 Inpatient bleed: intracranial haemorrhage, 0.038; gastrointestinal bleed, 0.45; other inpatient bleed, 0.45	No/outpatient bleeds: utility in general UK population and TOBIT model [33] built based on patient-level data from avatrombopag Phase 3 trial [26] Inpatient bleeds: romiplostim NICE submission [21] and eltrombopag NICE submission [22]
Disutilities, mean (SE)	TPO-RAs: 0.10 (0.025) Concomitant and rescue therapies: see Table S1	Romiplostim NICE submission [21]; eltrombopag NICE submission [22]; data on file; TOBIT model [33]
**Other.**		
Proportion of patients using concomitant ITP medication	Non-response state: 44.9% Response state: 35.9% ^i^ Additional information on concomitant medication: see Table S1	Avatrombopag Phase 3 trial [26]
Proportion of patients using rescue medication	Platelets ≥ 50 × 10^9^/L: 3.0% Platelets < 50 × 10^9^/L: 22.0%	Eltrombopag NICE submission [22]
Serious TRAEs	See Table S3 [45,46]	Romiplostim NICE submission [21]
Mortality	ITP mortality: intracranial haemorrhage, 13.2% (95% CI, 9.8–16.6%); gastrointestinal bleed, 4.6% (2.7–6.4%); other inpatient bleed, 1.7% (1.4–2.0%) All-cause mortality: variable, depending on age and sex	ITP mortality: Danese et al., 2009 [34] All-cause mortality: life tables from the Office for National Statistics [35]; age and sex distribution based on those observed in avatrombopag Phase 3 trial [26]
Dosing—rescue and concomitant ITP therapies	See Table S4 [47,48]	See Table S4 [47,48]

^a^ The probability of durable response values for eltrombopag and romiplostim are the relative probabilities of a treatment response versus avatrombopag, derived using Bayesian or frequentist analysis of the placebo-adjusted response rates from Phase 3 clinical trials, as summarised in durable response data row. ^b^ Assumed minor bleeds were self-treated without associated costs. ^c^ The distribution of bleeding types data are the relative distributions of bleed types amongst patients with bleeds requiring hospitalisation (e.g., of the 4.3% of patients with platelets levels < 50 × 10^9^/L assumed to have an inpatient bleed, 19% would have an intracranial bleed, 19% would have a gastrointestinal bleed and 63% would have another type of bleed). ^d^ An increased NHS tariff was used to take into account the higher potential costs for managing bleeds in patients with ITP compared with the general population. ^e^ Supplementary File S1 [29,30,31,42,49,50,51,52]. ^f^ Mean dose reported in long-term extension study of Phase 3 trial [32]. ^g^ Assumptions for home administration of romiplostim (which incurs no administration costs): 0% of patients for the first cycle (4 weeks) and then 72.3% of patients from cycle 2 onwards; this is based on data from Kuter et al., 2010 [32]. ^h^ Each month during treatment, patients were assumed to receive one haematologist consultation, and two laboratory tests, full blood count and one biochemistry assessment. ^i^ 20% reduction versus non-response state. CI: confidence interval; HRG: Healthcare Resource Group; ITP: immune thrombocytopenia; NC: not calculated; NHS: National Health Service; NICE: National Institute for Health and Care Excellence; NMA: network meta-analysis; SE: standard error; SmPC: summary of product characteristics; TPO-RA: thrombopoietin receptor agonist; TRAE: treatment-related adverse event.

#### 2.3.2. Bleeding Events

Bleeding events were characterised as minor, outpatient (not requiring hospitalisation) and inpatient (requiring hospitalisation: intracranial, gastrointestinal and other inpatient bleeds). Minor bleeds were assumed to be self-treated without associated costs. Based on the NICE submission for eltrombopag [22], the risk of an outpatient or inpatient bleeding event was assumed to be higher with a platelet count < 50 × 10^9^/L (Table 1) and therefore dependent on type of treatment. It was also assumed that patients with an inpatient bleed also had a risk of ITP-related death.

#### 2.3.3. Administration of Concomitant ITP Medication

Concomitant ITP medications considered in the model (danazol, azathioprine, cyclosporine, etamsylate, dexamethasone, prednisolone and prednisone) were those used in the Phase 3 avatrombopag trial (i.e., the most recent Phase 3 TPO-RA trial) [26] (Table S1). A reduction in concomitant ITP medication could only occur during the ‘Treatment, response’ health state. The probability of concomitant ITP medication was dependent on treatment response health state (not treatment type) and was higher in the ‘Treatment, no response’ and ‘No active treatment’ states than the ‘Treatment, response’ state.

#### 2.3.4. Administration of Rescue Therapy

Based on the NICE submission for eltrombopag [22], rescue therapy was reserved for emergency use only, i.e., an urgent need to increase platelet count. Rescue therapies considered in the model were IVIg, intravenous corticosteroids and platelet transfusion (Table S1).

#### 2.3.5. Treatment-Related Adverse Events

The results of the NMAs which reported comparative data for the individual TPO-RAs showed that there were no statistically significant differences between avatrombopag, eltrombopag and romiplostim for the incidence of AEs/TRAEs [12,13,14,16,19]; consequently, the model assumed that the three TPO-RAs had the same profile [46]. The incidences of serious and other TRAEs for TPO-RAs, concomitant and rescue therapies were adopted from the romiplostim NICE single technology appraisal [45] (Table S3).

#### 2.3.6. Mortality

ITP-related mortality was based on mortality rates associated with intracranial bleed (13.2%), gastrointestinal bleed (4.6%) and other inpatient bleed (1.7%) [34] (Table 1). All-cause mortality was based on 2019 UK national statistics [35]. The average age and sex distribution for patients with ITP was derived from the Phase 3 avatrombopag trial [26] (which was consistent with the Phase 3 trials of romiplostim [28] and eltrombopag [27]).

#### 2.3.7. Health-State Utilities

Utility values (which conventionally range between 0 [representing death] and 1 [representing perfect health]) in patients with or without bleeding events were estimated from EuroQol five-dimension (EQ-5D) questionnaire index score data in the UK general population and patient-level data from the Phase 3 avatrombopag trial [46] (Table 1). EQ-5D is a generic measure of quality of life routinely used in cost-effectiveness analyses, while the patient-level data allowed the use of utility values that correspond to the model health states. Disutilities associated with serious TRAEs for TPO-RAs, concomitant ITP medication and rescue therapy were based on the NICE submission for eltrombopag [37]. Utilities and disutilities of all heath states were assumed to be the same for all TPO-RAs (Table 1).

#### 2.3.8. Costs and Resource Utilisation

The model included costs incurred for the acquisition and (if appropriate) administration costs for treatment (TPO-RAs and concomitant ITP medication) and the management of bleeding events (Table 1). The costs for managing bleeding events were also included in the model. As bleeding cost data are limited to NHS tariffs for the general population, increased NHS tariffs were included in the base-case model, on the basis of data from a qualitative study in Europe suggesting that patients with ITP are likely to require substantial healthcare resources [30]. Details of calculation of increased NHS tariffs are provided in Supplementary File S1 [29,30,31,42,49,50,51,52]. The impact of varying bleeding management costs was evaluated in scenario analyses. Costs for healthcare resource utilisation (HCRU) required to manage bleeds in the model included emergency-room admission; hospital stay; emergency surgery; ambulance use; diagnostic imaging; and blood tests. Follow-up costs included consultation and biochemistry/blood tests.

### 2.4. Model Outputs

The model estimated the following outcomes: incremental quality-adjusted life-years (QALYs), incremental life-years, incremental costs and incremental cost-effectiveness ratio (ICER).

### 2.5. Model Validation

Model validation was performed using three elements. Face validation was performed to ensure this model’s specification was aligned to TPO-RA submissions to NICE [37,45,46] and other published cost-effectiveness models for TPO-RAs [23,24]. Internal validation consisted of quality checks conducted for model codes, model inputs (source comparisons and intermediate calculations) and model outputs. Cross-validation consisted of comparing model results with those obtained for other TPO-RA models [21,22,23,24].

### 2.6. Model Assumptions

Several key assumptions were made in the model, as summarised in Table S5 [12,13].

### 2.7. Sensitivity and Scenario Analyses

Probabilistic sensitivity analysis (PSA) was performed to assess the level of parametric uncertainty associated with model point estimate input values. Model input parameters subject to uncertainty were randomly sampled within their plausible bounds (i.e., the range of possible values that a variable can assume) to record separate results of cost-effectiveness by running the model multiple times. The robustness of the model was tested by a one-way deterministic sensitivity analysis (DSA), where one parameter or model assumption was varied (+/− 20.0% of the point estimate for most parameters) separately, whilst the other parameters retained their base-case values (Table S6 [25,26,28,34]). The model was run for a total of 1000 iterations to assess the effect of each parameter change on the model outcomes and the ICER, and the results were presented using a tornado diagram.

Five scenario analyses were conducted. The first scenario applied alternative probabilities of durable platelet response (Table 1) derived from an indirect treatment comparison conducted using a frequentist framework and the same zero-correction factor as the base-case analysis (Table S2). In the second scenario analysis, patients who did not respond to TPO-RA treatment (or whose initial response was not maintained) could receive up to three further lines of active (non-TPO-RA) treatment, the distribution of which was based on a qualitative study in Europe [30] (Table S7). As part of this scenario analysis, it was assumed that patients who were refractory to all four lines of therapy had an increased (2-fold) rate of an inpatient bleed (based on the NICE submission for eltrombopag [22]). The next two scenarios applied alternative costs of managing bleeding events based on the standard NHS tariff [29] or the results of the European qualitative study [30] (Table 1). In the final scenario, the time period over which treatment response was assessed was reduced from 28 weeks to 12 weeks.

## 3. Results

### 3.1. Base-Case Analysis

Over a lifetime horizon, avatrombopag was associated with higher QALYs (10.979) compared with eltrombopag and romiplostim (10.085 and 10.628, respectively), producing incremental QALY gains of 0.894 and 0.351, respectively (Table 2). Avatrombopag was associated with higher total costs (GBP 319,334) compared with eltrombopag (GBP 313,987), producing a cost increment of GBP 5347, and lower total costs compared with romiplostim (GBP 406,361), producing a cost saving of GBP 87,027 (Table 2). A breakdown of the comparative costs for avatrombopag versus eltrombopag and romiplostim are shown in Table 3. Based on the incremental QALYs and costs, avatrombopag was cost-effective versus eltrombopag (ICER GBP 5982 per QALY) and dominated (more effective and less expensive) romiplostim (Table 2).

### 3.2. Sensitivity Analyses

**Figure 2:**
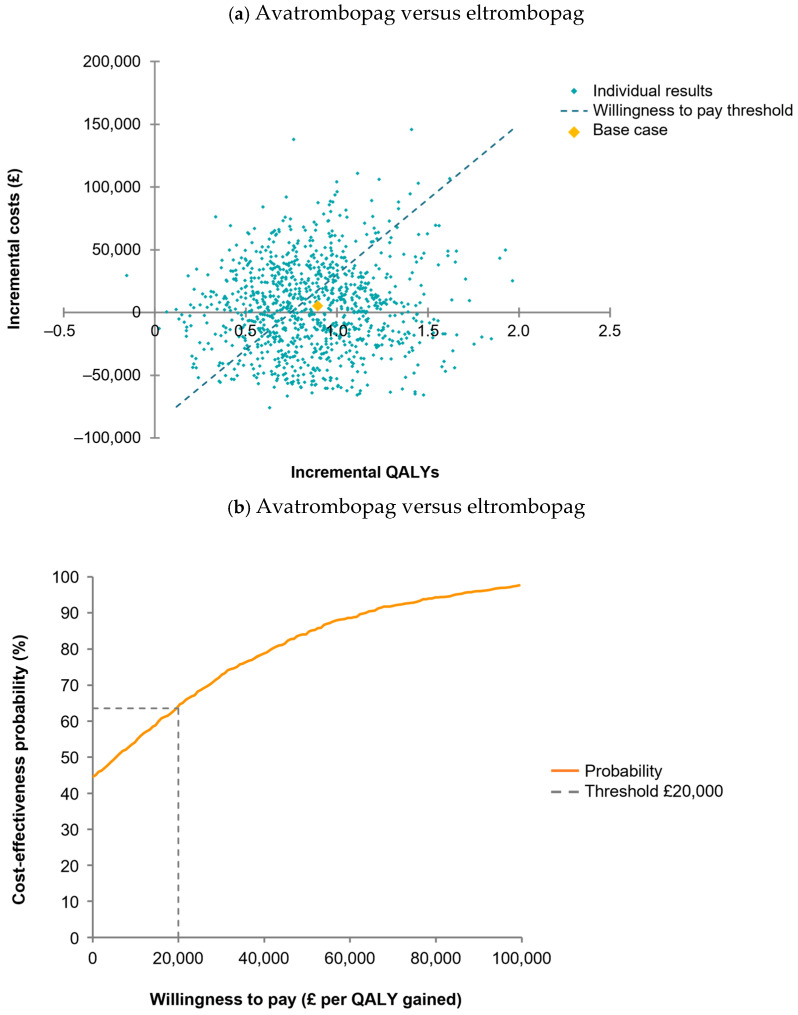

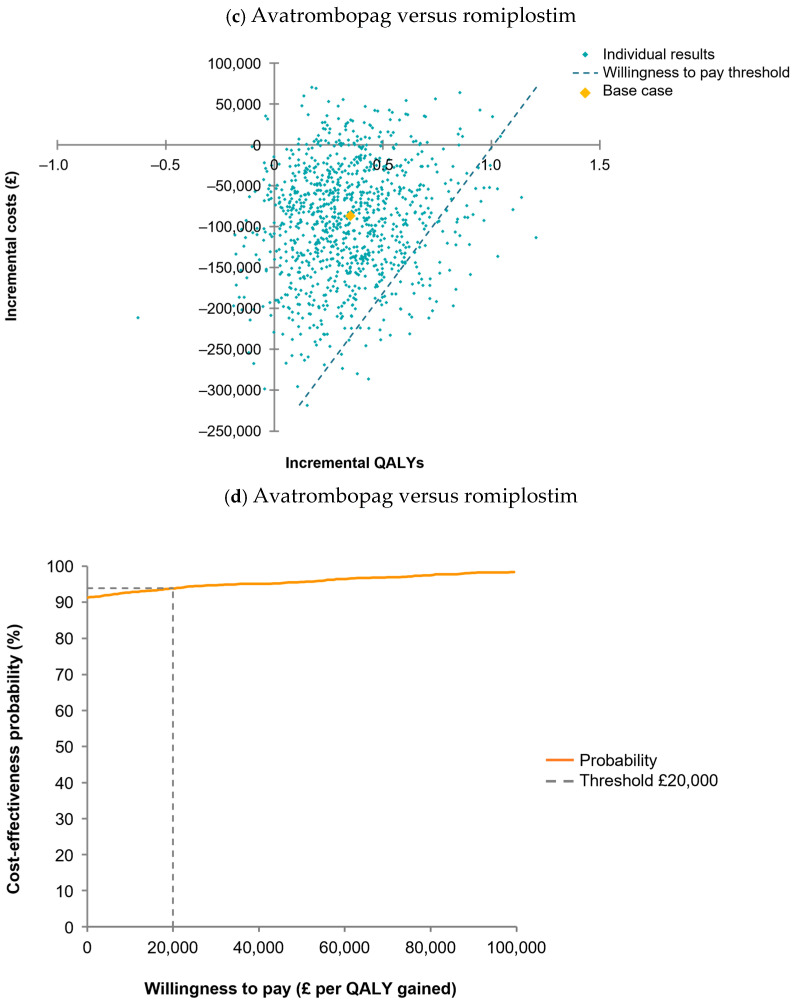
Probabilistic sensitivity analysis (**a**,**c**) and cost-effectiveness acceptability curves (**b**,**d**). The cost-effectiveness scatter plot presented on a cost-effectiveness plane (shown in (**a**,**c**)) consist of four quadrants, where the *x*-axis represents incremental QALYs with avatrombopag (versus eltrombopag and romiplostim) and the *y*-axis represents the incremental costs. In the eltrombopag comparison, the base-case value (orange dot) is located in the top right quadrant showing that avatrombopag was cost-effective (more effective and more costly). In the romiplostim comparison, the base-case value (orange dot) is located in the bottom right quadrant showing that avatrombopag was dominant (more effective and less costly). The cost-effectiveness scatter plot also shows the results of 1000 PSA iterations, represented by green dots, which nearly all fall within the cost-effective and dominant quadrants (avatrombopag versus eltrombopag) or the dominant quadrant (avatrombopag versus romiplostim). The cost-effectiveness acceptability curves (shown in (**b**,**d**)) show cost-effectiveness thresholds on the *x*-axis, the probability that avatrombopag will be cost-effective for different thresholds on the *y*-axis, and the proportion of the 1000 iterations that are cost-effective for a particular cost-effectiveness threshold (orange line; not a cumulative distribution). At a threshold of GBP 20,000/QALY (dashed line), the probabilities that avatrombopag was cost-effective were 63.6% (versus eltrombopag) and 93.9% (versus romiplostim). PSA, Probabilistic sensitivity analysis; QALY: quality-adjusted life-year.

The PSA results are presented in Figure 2. At a cost-effectiveness threshold of GBP 20,000 per QALY, the probabilities that avatrombopag was cost-effective or dominant compared with eltrombopag were 63.6% and 44.5%, respectively. The probabilities that avatrombopag was cost-effective or dominant compared with romiplostim were 93.9% and 85.9%, respectively. In the DSA, avatrombopag dominated or was cost-effective versus eltrombopag across all parameters varied except for the ‘upper bound estimate’ of avatrombopag dose (ICER of GBP 21,356 per QALY) (Figure S1). Avatrombopag was dominant versus romiplostim across all the parameters varied in the DSA.

### 3.3. Scenario Analyses

#### 3.3.1. Bayesian Framework

Avatrombopag remained cost-effective versus eltrombopag when using the standard NHS tariff for bleeding management costs, up to three subsequent lines of treatment, and a treatment response period of 12 weeks. Avatrombopag dominated eltrombopag when using bleeding management costs based on data from the qualitative study (Table S8a). In addition, avatrombopag remained dominant over romiplostim across all these scenarios (Table S8b).

#### 3.3.2. Frequentist Framework

Avatrombopag was dominant over eltrombopag in all five scenario analyses (Table S8a). Compared with romiplostim, avatrombopag was associated with lower incremental QALYs and lower incremental costs, resulting in ICERs ranging from GBP 747,313/QALY to GBP 858,704/QALY, indicating that avatrombopag is cost-effective versus romiplostim (Table S8b).

## 4. Discussion

Avatrombopag, eltrombopag and romiplostim are the recommended TPO-RA treatment options for patients with ITP who have not responded adequately to first-line treatment. The base-case analysis of the economic model used here demonstrates that avatrombopag is a cost-effective treatment option compared with eltrombopag and a dominant treatment option (more effective and less expensive) compared with romiplostim. Linked to the previously published efficacy and safety data for avatrombopag [26], the results of this cost-effectiveness analysis support that avatrombopag provides a valuable treatment option for adults with ITP.

The robustness of the base-case findings is supported by the results of the sensitivity and scenario analyses. The DSA showed that almost all model parameters appeared to be drivers of cost-effectiveness/dominance for avatrombopag versus eltrombopag and romiplostim. Several of the scenario analyses varied the costs associated with the management of bleeding, reflecting the importance of bleeding episodes as the primary cause of long-term morbidity and mortality in patients with ITP [53]. Avatrombopag remained cost-effective versus eltrombopag when the alternative bleeding costs were based on the standard NHS tariff and became dominant when based on a recent European qualitative study. Avatrombopag remained dominant over romiplostim in both analyses. When the frequentist approach with a proportional continuity correction for zero events was used for indirect comparison between considered treatments, avatrombopag was dominant versus eltrombopag and it was cost-effective versus romiplostim. As expected, despite some differences in relative efficacy resulting from the frequentist approach (versus the Bayesian approach), the conclusions concerning cost-effectiveness of avatrombopag from this scenario were largely consistent with the base case. In the other scenario analyses, changing the treatment response period to 12 weeks and including the use of up to three additional therapy lines had little impact on the cost-effectiveness results and avatrombopag remained dominant or cost-effective versus eltrombopag and romiplostim.

Markov modelling is a well-accepted methodology for conducting pharmacoeconomic evaluations, and is particularly suitable for diseases that involve an ongoing risk over time (e.g., risk of haemorrhage) [54]. The model structure used is consistent with the established previously described cost-effectiveness analyses of romiplostim versus eltrombopag and standard of care from Irish and UK healthcare perspectives [24], and eltrombopag versus romiplostim from the perspective of the UK NHS [55]. In addition, model validation demonstrated that the results of our base-case analysis were comparable to the cost-effectiveness model results presented in the literature.

Other differences in the three TPO-RAs were not captured in the model. For example, avatrombopag and eltrombopag are administered as a once-daily tablet, while romiplostim is administered as a weekly subcutaneous injection. Romiplostim requires weekly dose adjustment by the managing physician, typically in the clinic, until a stable dose (≥50 × 10^9^/L for ≥4 weeks without dose adjustment) is achieved [56]. Subsequently, the patient could potentially be allowed to self-administer at home. As such, oral administration may be associated with a utility gain, which, for simplicity, was not included in the current model. Avatrombopag is the only oral option that can be administered without dietary restrictions, which may improve adherence. Patient choice in route of administration remains paramount—some patients will prefer a once-a-week administration and others may prefer a daily tablet. Overall, TPO-RAs represent a paradigm shift in the second-line treatment of ITP, and there is evidence that some patients may be switched to an alternative TPO-RA as third-line treatment before the use of other treatment approaches such as invasive splenectomy [30,57,58].

To the best of the authors’ knowledge, this paper represents the first published account of the cost-effectiveness of avatrombopag in patients with ITP. The cost-effectiveness of eltrombopag versus romiplostim has been evaluated previously [55], but avatrombopag was not included in the model as it was not approved at the time. A recent cost-effectiveness analysis compared six treatment strategies involving splenectomy, rituximab and TPO-RAs (as a class) for ITP from the US healthcare perspective [59]. It concluded that splenectomy followed by rituximab and then TPO-RAs represented the cost-effective treatment strategy. However, according to international consensus, based on patient acceptability and post-surgical complications, splenectomy is now recommended only after failure of medical therapies and depending on patient age and comorbidities [7]. A budget impact analysis assessed the introduction of avatrombopag, in addition to romiplostim and eltrombopag, for the management of adults with chronic primary ITP refractory to other treatments (n = 1741) over a 3-year period within the Italian NHS [60]. This study found that the introduction of avatrombopag was associated with a total cost saving of €6,083,231, with savings in both drug therapy and monitoring costs, compared with the reference treatment scenario without avatrombopag. In contrast, a real-world retrospective study of adults with chronic ITP in the USA treated between June 2018 and December 2021 suggested that avatrombopag was associated with higher incremental total mean per patient costs of care (including primary drug therapy, rescue therapy, and management of AEs and thromboembolic events) of $38,624 versus eltrombopag and $22,568 versus romiplostim [61]. However, the differences in total mean costs per patient were not statistically significant, and drug acquisition costs appeared to be the primary driver of the overall cost of care for each of the treatments (accounting for approximately 97% of the total cost). In view of its retrospective observational design, limitations of this study, as acknowledged by the authors, include potential bias as a result of unmeasured confounding variables and difficulties assessing and quantifying the severity of bleeding events using established grading scales.

A key data source for the current model was the odds ratios for durable platelet response based on a recent NMA [13], which was used in the absence of head-to-head trials comparing avatrombopag, eltrombopag and romiplostim. This NMA was used in the health-technology assessment by NICE [62] and employed a comprehensive methodology involving an SLR and several Phase 3 double-blind randomised clinical trials providing evidence for avatrombopag (n = 2), eltrombopag (n = 1) and romiplostim (n = 2). Another strength is that the model intended to be representative of clinical practice by using bleeding cost data based on the significant healthcare resources required for patients with ITP, and due to the consideration of concomitant medication and rescue therapy for ITP. Other strengths include the assessment of long-term outcomes via the lifetime horizon; use of durable response for at least 6/8 weeks to account for transient platelet counts < 50 × 10^9^/L that may occur in clinical practice; and the determination of durable response rates using two alternative and valid methods (Bayesian and frequentist frameworks).

A key limitation of the analysis was the lack of data on the cost of managing bleeding events in patients with ITP. NHS tariffs provide information on the cost of managing these events in the general population only. Patients with ITP have a pathophysiological reduction in platelet counts compared with the general population and are likely to have longer bleed duration, longer time to increased platelet count and stabilised bleeding, worse severity of bleeds and greater resource utilisation; as such, standard NHS tariffs are likely to underestimate those in patients with ITP. Costs in the base-case analysis were therefore based on an increased NHS tariff with the aim that the results may more closely reflect clinical practice in a population of patients with ITP. However, we acknowledge that evidence supporting an increased NHS tariff in patients with ITP have not been derived from a validated study. Scenario analysis based on the standard NHS tariffs did not change the overall conclusions of the model, i.e., avatrombopag was cost-effective versus eltrombopag and dominated romiplostim. A qualitative study, which collected opinions from >100 ITP treaters across Europe, suggested that patients with ITP and moderate bleeding events are hospitalised for an average of 6–11 days, and those with severe bleeds are hospitalised 10–20 days, supporting the HCRU burden of patients with ITP [30]. In an additional scenario analysis using bleeding management costs estimated based on this qualitative study, avatrombopag dominated both eltrombopag and romiplostim. It is acknowledged that this scenario analysis has limitations as it is based on qualitative opinion-based data on ITP management, rather than evidence-based costs. However, a NICE technology appraisal committee presented with these data recognised that there might be additional resources associated with managing bleeding events not covered by the NHS reference costs [62]. Another limitation of the analysis was the reliance, in the absence of more recent data, on model inputs from the eltrombopag model for NICE, which was submitted in 2012. In addition, the average TPO-RA dose was based on the summary of product characteristics (eltrombopag and avatrombopag) or historical clinical trial data (romiplostim), which may not reflect current real-world clinical management and may result in different costs dependent on specific clinical practices. Furthermore, we assumed that 72.3% of patients administered romiplostim at home, but the proportion may vary across countries and likely impact the overall cost of this treatment.

A further important limitation was the lack of head-to-head efficacy and safety data for TPO-RAs and uncertainty related to an indirect treatment comparison and possible underpowering in the trials, particularly the placebo arms. The clinical trials for the TPO-RAs included in the NMA were selected according to the homogeneity of their clinical data. However, they were conducted during different time frames and may therefore involve different patient populations. They had some methodologic differences and long-term outcomes were not assessed. In addition, we assumed no differences in the incidence of TRAEs between the TPO-RAs, based on the evidence of no statistically significant differences in the relevant NMAs [12,13,14,16,19], but acknowledge TRAEs rates may differ in routine clinical practice. Further, the TPO-RAs have some similarities in TRAEs (e.g., headache and fatigue) but the safety profiles do not completely overlap, such as an increased risk of severe hepatotoxicity with eltrombopag [44].

Another limitation is that treatment response (e.g., cut-off platelet levels of ≥50 × 10^9^/L) was defined, by necessity, according to the parameters set in the clinical trials, and this may not reflect current clinical practice (e.g., a threshold of <20–30 × 10^9^/L). Similarly, the dose required to achieve and/or maintain a treatment response in routine practice may differ from the dose used in clinical trials. Furthermore, some of the assumptions of the model may not exactly reflect clinical practice, for example, the model had a lifetime horizon but patients may discontinue treatment (for different reasons) prior to death; however, these are unlikely to affect the results as they apply to all three TPO-RAs. Using real-world data for model inputs (e.g., to compare clinical data or costs) may reflect clinical practice more closely, but as discussed above, is also subject to certain challenges, including the inconsistent study designs and accuracy of outcome measure assessment [63]. Additional limitations of the current study include an inability of the model to account for confidential commercial discounts drug acquisition (net) costs (applicable to all three TPO-RAs) and the use of assumptions for some parameters when they could not be supported by published data.

## 5. Conclusions

The results of this evidence-based model, from the perspective of the UK NHS, indicate that avatrombopag is a cost-effective treatment strategy versus eltrombopag and a dominant treatment strategy versus romiplostim for adults with ITP who have an insufficient response to previous treatment. The findings of this comparative assessment of cost-effectiveness may help inform clinical and healthcare policy decisions regarding the available second-line treatment options for the management of patients with ITP.

## Figures and Tables

**Table 2:** Base-case analysis results for avatrombopag versus eltrombopag and romiplostim.

	Total QALYs	Incremental QALYs	Total LYs	Incremental LYs	Total Cost (GBP)	Incremental Cost (GBP)	ICER (Cost per QALY)	Cost per LY
Avatrombopag	10.979	–	16.199	–	319,334	–	–	–
Eltrombopag	10.085	−0.894	15.252	−0.947	313,987	−5347	5982	5649
Romiplostim	10.628	−0.351	15.827	−0.372	406,361	87,027	Dominant	Dominant

ICER: incremental cost-effectiveness ratio; LY: life-year; QALY: quality adjusted life year.

**Table 3:** Cost breakdown for avatrombopag versus eltrombopag and romiplostim (base-case analysis).

	Avatrombopag	Eltrombopag	Romiplostim
Total costs ^a^	**319,334**	**313,987**	**406,361**
Treatment costs	159,505	138,559	236,127
Active treatment	72,778	43,254	146,032
Rescue therapy	72,863	81,769	76,359
Concomitant ITP medications	13,865	13,536	13,736
Treatment administration costs	32,080	36,002	37,901
Active treatment	0	0	4281
Rescue therapy	32,080	36,002	33,620
Concomitant ITP medications	0	0	0
Monitoring cost	34,461	32,447	33,671
Bleeding costs	93,288	106,979	98,663
Minor bleed	0	0	0
Outpatient bleed	36,147	40,370	37,805
Inpatient bleed type	57,141	66,609	60,858
Intracranial haemorrhage	16,600	19,351	17,680
Gastrointestinal bleed	12,969	15,117	13,812
Other inpatient bleed	27,572	32,140	29,366

^a^ Totals and subtotals may include differences due to rounding of decimals. ITP: immune thrombocytopenia.

## Data Availability

The datasets generated during and/or analysed during the current study are available from the corresponding author on reasonable request.

## Supplementary Materials

The following supporting information can be downloaded at: https://www.mdpi.com/article/10.3390/jmahp13020011/s1, Supplementary File S1; Table S1: Data sources and values for model inputs related to concomitant and rescue therapies; Table S2: Odds ratios for durable platelet response for TPO-RAs versus placebo; Table S3: Treatment-related adverse event rates; Table S4: Dosage of concomitant ITP and rescue medications; Table S5: Key assumptions of the model; Table S6: Deterministic sensitivity analyses inputs; Table S7: Distribution of later-line treatments after non-response or loss of response to treatment I (avatrombopag, eltrombopag or romiplostim); Table S8: Results of scenario analyses; Figure S1: Tornado chart for avatrombopag versus eltrombopag (deterministic sensitivity analysis); and References.
