# Supplementary material for: The Cost-Effectiveness of Avatrombopag Versus Eltrombopag and Romiplostim in the Treatment of Patients with Immune Thrombocytopenia in the UK
Source: J Mark Access Health Policy. 2025 Mar 24;13(2):11. doi: 10.3390/jmahp13020011 (PMC12015888; doi:10.3390/jmahp13020011)
Supplement: Supplementary file 1 [file jmahp-13-00011-s001.zip › jmahp-3287194-supplementary.pdf]

SUPPLEMENTARY MATERIALS

The Cost-Effectiveness of Avatrombopag Versus Eltrombopag and Romiplostim in the Treatment of Patients with Immune Thrombocytopenia in the UK

Nichola Cooper [1,*], Sebastian Guterres [2], Michał Pochopień [3], Koo Wilson [4], Sam James [2], Mondher Toumi [5], Anna Tytuła [6], Carly Rich [4] and Daniel Eriksson [4]

[1] Faculty of Medicine, Department of Immunology and Inflammation, Imperial College London, London, UK
[2] Sobi, Cambridge, UK
[3] Assignity, Kraków, Poland
[4] Sobi, Stockholm, Sweden
[5] Department of Public Health, Aix-Marseille University, Marseille, France
[6] Health Economics and Outcomes Research Department, Putnam PHMR, Kraków, Poland
* Correspondence: n.cooper@imperial.ac.uk

Supplementary Appendix 1

Calculation of Bleeding Costs: Increased NHS Tariff (Base-Case)

It was assumed that the duration of different bleeds in patients with immune thrombocytopenia (ITP) tends to be longer in comparison to the general population, as it takes a relatively long time to increase the patient's platelet count and stabilise bleeding. Moreover, the severity of bleeds in patients with a low platelet count tends to be greater. It was therefore considered reasonable to uplift National Health Service (NHS) reference costs for bleeding events to account for additional resources associated with bleeding in patients with ITP.

Two approaches for uplifting NHS reference costs are presented in the following table: 1) a weighted average of NHS unit costs associated with different complication and comorbidity (CC) scores from NHS reference costs; (2) a selection of the highest unit costs for each type of bleed corresponding to those with the highest CC score.

The authors selected the highest unit costs (2) in the base case based upon clinical understanding of the magnitude of additional resources associated with treating bleeding in patients with ITP relative to the general population. The average weighted costs (1) (i.e., standard NHS tariff) were included in a scenario analysis.

**Type of bleed**	**Weighted average**		**Unit cost**	
	**Cost (£)**	**Source (NHS reference cost 2019/20)[a]**	**Cost (£)**	**Source (NHS reference cost 2019/20)[a]**
Outpatient bleed	459.65	Weighted average FD03F-FD03H	493.74	FD03F
Gastrointestinal bleed	3091.79	Weighted average FD03A to FD03E	5502.62	FD03A
Intercranial haemorrhage	4690.02	Weighted average AA23C to AA23G	7044.18	AA23C
Other inpatient bleed	2890.37	Weighted average, FD03B and FD03E	3485.00	FD03B

[a][1].

NHS: National Health Service.

Calculation of Bleeding Costs: Qualitative study (Scenario Analysis)

Health states in the model are driven by platelet response, which influences costs associated with bleeding events, use of rescue therapy and concomitant ITP medication usage. Costs associated with bleeding events have been informed by a qualitative study in Europe [2] that explored different elements of healthcare resource utilisation for outpatient and inpatient bleeds, including life-threatening bleeds (intracranial haemorrhage); details are provided in the table below. Minor bleeds were assumed to be self-treated and have no associated costs.

Utilisation inputs used in bleed managements by category of bleed

Bleed type	Outpatient bleeds Utilisation of item per event	Outpatient bleeds Utilisation in event %	Inpatient bleed (GI/other) Utilisation of item per event	Inpatient bleed (GI/other) Utilisation in event %	Inpatient bleed (ICH) Utilisation of item per event	Inpatient bleed (ICH) Utilisation in event %
**ER/hospital stay**						
ER admission	1	100	1	100	1	100
ICU bed					4	100
Ward bed			6	100	7	100
Outpatient care	1	100				
Emergency surgery						
Neurosurgery			1	10	1	30
GI surgery			1	40	1	70
**Other**						
Ambulance			1	60	1	100
Diagnostic imaging and blood tests						
CT	1	60	1	100	1	100
MRI	1	60	1	100	1	100
Blood work	1	100	1	100	1	100
Follow-ups						
Haematologist Follow-up	3	100	6	100	12	100
**Therapies (total)**						
IVIg and corticosteroids						
IVIg[a]	1	40	2	100	2	100
Methylprednisolone[b] (140 mg × 3 days)	3	100	3	100	3	100
Platelet transfusion						
Platelets (cost per day)			5	100	15	100
**Other**						
Factor VIIa, recombinant[c,d]			1	40	1	60

[a]70.8 g (1 g/kg for average adult); [b]140 mg × 3 days; [c]5.3 mg (75 µg/kg for average adult); [d]Based on data from Pogna et al. 2021 [2]; the data indicated treatment used for patients with haemophilia with inhibitors – this may not be routinely used for patients with ITP in routine clinical practice.

CT: computed tomography, ER: emergency room, GI: gastrointestinal, ICH: intracranial haemorrhage, ICU: intensive care unit, ITP: immune thrombocytopenia, IVIg: intravenous immunoglobulin, MRI: magnetic resonance imaging.

Each element of resource use is assigned a unit cost from established UK sources, presented in the following table.

Unit costs of resources used in bleeding management

Category	Value (£)	Source
**ER/hospital stay**		
ER admission	160	NHS reference cost 2019/2020 (cost of attendance) [3]
ICU bed (daily cost)	1364	NHS reference cost 2019/2020 [3]. Average daily cost of intensive care bed
ICU bed (level 3 daily cost)	1932	Average cost of level 3 intensive care bed day [4]
ICU bed	1648	Average of above
Ward bed (average daily cost)	310	University Hospitals Birmingham Trust 2018 [5]
Ward bed (bed day cost)	346	NHS reference cost 2019/2020 [3]
Ward bed	328	Average of above
Outpatient visit	742	NHS reference cost 2019/2020 [3]
**Emergency surgery**		
Neurosurgery	2383	NHS reference cost 2019/20, intermediate intracranial procedures, 19 years and over, with CC score 0–1 [1]
GI surgery	2230	NHS reference cost 2019/20, gastrointestinal bleed with a single intervention, with CC score 0–4 [1]
**Other**		
Ambulance		
Incidents attended	1,140,980	London Ambulance Service NHS Trust 2018/2019 [6]
Total operating expenses	378,154,000	London Ambulance Service NHS Trust 2018/2019 [6]
Ambulance (cost per incident)	331	Average of above
**Diagnostic imaging and blood tests**		
CT	83	NHS reference cost 2019/20[a] [1]
MRI	157	NHS reference cost 2019/20[a] [1]
Blood work	26	NICE 2015, full blood count [7]
**Follow-ups**		
Haematologist follow-up (first visit)	275	NHS reference cost 2019/20, first single professional attendance [1]
Haematologists follow-up (every other visit)	125	NHS reference cost 2019/20, follow-up attendance, single professional [1]
**Therapies**		
IVIg and corticosteroids		
IVIg (70.8 g, 1 g/kg for average adult)	3823.20	BNF Medicines Complete 2021 [8]
Methylprednisolone (140 mg × 3 days)	2.69	BNF Medicines Complete 2021 [8]
Platelet transfusions		
Platelets (price per day)	186.86	NHS reference cost 2019/20 [1]
**Other**		
Factor VIIa, recombinant (5.3 mg, 75 μg/kg for average adult)	2783.56	BNF Medicines Complete 2021 [8]

[a]Tariff includes cost of reporting.

BNF: British National Formulary, CC: complication and comorbidity, CT: computed tomography, ER: emergency room, GI: gastrointestinal, ICU: intensive care unit, IVIg: intravenous immunoglobulin, MRI: magnetic resonance imaging, NHS: National Health Service, NICE: National Institute for Health and Care Excellence.

Combining resource utilisation and unit costs provides a cost of management per bleeding event for outpatient bleeds and inpatient bleeds, including life-threatening bleeds (intracranial haemorrhage). The costs per bleeding event used in the model are presented in the table below.

Cost of bleeding event management by bleed type used in model (£)

**Bleed type**	**Outpatient bleed (£)**	**Inpatient bleed; gastrointestinal/other (£)**	**Inpatient bleed; intracranial haemorrhage (£)**
**ER/hospital nights**			
ER admission	160	160	160
ICU bed			6592
Ward bed		1968	2296
Outpatient care	742		
**Emergency surgery**			
Neurosurgery		238	715
Gastrointestinal surgery		892	1561
**Other**			
Ambulance		199	331
Diagnostic imaging and blood tests			
CT	50	83	83
MRI	94	157	157
Blood work	26	26	26
Follow-up			
Haematologist follow-up	525	900	1650
**Therapies (total)**			
IVIg and corticosteroids			
IVIg (70.8 g, 1 g/kg for average adult)	1529	7646	7646
Methylprednisolone (140 mg × 3 days)	8	8	8
Platelet transfusion			
Platelets (price per day)		934	2803
**Other**			
Factor VIIa, recombinant (5.3 mg, 75 µg/kg for average adult)		1113	1670
**Total**[a]	**3134**	**14,325**	**25,699**

[a]Totals may include differences due to rounding of decimals.
CT: computed tomography, ER: emergency room, ICU: intensive care unit, IVIg: intravenous immunoglobulin, MRI: magnetic resonance imaging.

Table S1 Data sources and values for model inputs related to concomitant and rescue therapies[a]

Input	Value	Source
Proportion of patients using treatment[b]	Prednisone: 77%<br>Prednisolone: 9%<br>Dexamethasone: 9%<br>Etamsylate: 27%<br>Azathioprine: 5%<br>Cyclosporine: 5%<br>Danazol: 5%<br>IVIg: 33%<br>IV steroid: 56%<br>Platelet transfusion: 22%	Avatrombopag Phase 3 trial [9,10]
Time to response ($\geq 50 \times 10^9$/L)[c]	Azathioprine: 16 weeks (4 cycles)<br>Cyclosporine: 8 weeks (2 cycles)<br>Danazol: 16 weeks (4 cycles)	Romiplostim NICE submission [11]
Duration of response[c]	Azathioprine: 20 cycles<br>Cyclosporine: 16 cycles<br>Danazol: 147 cycles	Romiplostim NICE submission [11]
Serious AE disutilities, mean (SE)[c]	Azathioprine, cyclosporine, danazol: 0.40 (0.1)<br>All rescue medication (IVIg, IV steroid, platelet transfusion): 0.10 (0.025)	Eltrombopag Single Technology Appraisal [12]
Drug acquisition costs	Azathioprine: £2.57 (2.8 g)<br>Cyclosporine: £18.37 (750 mg)<br>Danazol: £36.32 (11.2 g)<br>Etamsylate: £9.00 (500 mg)<br>Dexamethasone: £49.00 (100 mg)<br>Prednisolone: £2.41 (140 mg)<br>Prednisone: £2.41 (140 mg)<br>IVIg: £50.00 (1 g)<br>IV steroid: £88.81 (1.2 g)	Etamsylate: Lloyds Pharmacy<br>Prednisone: Assumption<br>Others: NICE British National Formulary 2021
Therapy administration costs	IV steroids: £370.68 per administration<br>IVIg: £195.66 per infusion<br>Platelet transfusion: £889.66 per transfusion	NHS reference costs 2018/2019 [13]

[a]See Table 1 for key model inputs; [b]Concomitant medications were those ongoing at the time of avatrombopag initiation and/or started during avatrombopag treatment administration and up to 30 days after the last dose of treatment; [c]These were included in the model only when the medication was given as subsequent line of therapy or rescue therapy during subsequent lines of therapy (i.e., in the scenario analysis evaluating use of up to three lines of therapy in patients not responding to initial TPO-RA treatment). AE: adverse event, IV: intravenous, IVIg: intravenous immunoglobulin, NHS: National Health Service, NICE: National Institute for Health and Care Excellence, SE: standard error, TPO-RA: thrombopoietin receptor agonist.

Table S2 Odds ratios for durable platelet response for TPO-RAs versus placebo

Treatment comparison	OR (95% CI)	
	Bayesian framework	Frequentist framework
Avatrombopag vs placebo	102.8 (3.87–2796448.59)	26.91 (NC)
Eltrombopag vs placebo	14.27 (5.14–53.73)	10.60 (NC)
Romiplostim vs placebo	46.5 (2.13–181.7)	33.39 (NC)

Platelet response was defined as levels ≥50 × 10^9/L.

CI: confidence interval, NC: not calculated, OR: odds ratio, TPO-RA: thrombopoietin receptor agonist.

Table S3 Treatment-related adverse event rates

	Serious TRAEs	Other TRAEs	Source
Avatrombopag	3%	31%	Romiplostim Single Technology Appraisal [14]; Avatrombopag Single Technology Appraisal [15]
Eltrombopag	3%	31%	
Romiplostim	3%	31%	
Azathioprine[a]	15%	24%	
Cyclosporine[a]	15%	24%	
Danazol[a]	16%	35%	
Rescue – IVIg[a]	2%	0%	
Rescue – IV corticosteroid[a]	3%	70%	
Rescue – platelet transfusion[a]	0.011%	0%	

[a]These were included in the model only when the medication was given as subsequent line of therapy or rescue therapy during subsequent lines of therapy (i.e., in the scenario analysis evaluating use of up to three lines of therapy in patients not responding to initial TPO-RA treatment).

IV: intravenous, IVIg: intravenous immunoglobulin, TRAE: treatment-related adverse event, TPO-RA: thrombopoietin receptor agonist.

Table S4 Dosage of concomitant ITP and rescue medications

Drug	Treatment regimen dose	Frequency per 4 weeks cycle	Route	Duration days	Source
**Rescue medication**					
Rescue – IVIg	1000 mg/kg	28	Infusion	1.5	Provan et al. 2010 [16]
Rescue – IV steroid	1.25 mg/kg	28	Injection	3	
Rescue – platelet transfusion	2 platelet units	1	Transfusion	1	Assumption
**Concomitant ITP medications – without treatment response**					
Danazol	200 mg	84	Tablet	28	Provan et al. 2010 [16]
Azathioprine	1.5 mg/kg	28	Tablet	28	
Cyclosporine	5 mg/kg	28	Tablet	28	
Etamsylate	1500 mg	28	Tablet	15	Drug information etamsylate [17]
Dexamethasone	40 mg	4	Tablet	21	Provan et al. 2010 [16]
Prednisolone	1.25 mg/kg	21	Tablet	21	
Prednisone	1.25 mg/kg	21	Tablet	21	

ITP: immune thrombocytopenia, IV: intravenous, IVIg: intravenous immunoglobulin.

Table S5 Key assumptions of the model

Assumptions
The response to treatment had to be achieved within the first seven cycles after treatment initiation according to assumed time to response
Patients who achieved a response discontinued treatment based on the assumed treatment duration, although a proportion of these patients may continue treatment in clinical practice
The probabilities of bleeding and need for rescue therapy were dependent on the achieved platelet responses derived from the network meta-analysis
Data from the network meta-analyses indicated similar rates of treatment-related AEs across the three thrombopoietin receptor agonists [18,19]; it was therefore assumed the inclusion of treatment-related AEs would not lead to incremental changes in outcomes

AE: adverse event.

Table S6 Deterministic sensitivity analyses inputs

Settings & population				
	**Base-case value**	**Low value**	**High value**	**Source**
**General settings**				
Discount rate for health outcomes	3.5%	0.0%	5.0%	NICE reference case [20]
Discount rate for costs	3.5%	0.0%	5.0%	
**Population**				
Age	44.6	40.5568	48.6432	Avatrombopag Phase 3 trial [9]
Percentage of male	37%	29%	44%	Avatrombopag Phase 3 trial [9]
Weight (kg)	82.97	66.38	99.56	Avatrombopag Phase 3 trial [9]
Body area (m^2)	1.94	1.55	2.32	Estimations based on avatrombopag Phase 3 trial [9]
**Treatment**				
**Treatments in patients using rescue therapy**				
IVIg	33%	27%	40%	+/- 20.0%
IV steroids	56%	44%	67%	+/- 20.0%
Platelet transfusion	22%	18%	27%	+/- 20.0%
**Treatments in patients using concomitant ITP medications**				
Danazol	5%	4%	5%	+/- 20.0%
Azathioprine	5%	4%	5%	+/- 20.0%
Cyclosporine	5%	4%	5%	+/- 20.0%
Etamsylate	27%	22%	33%	+/- 20.0%
Dexamethasone	9%	7%	11%	+/- 20.0%
Prednisolone	9%	7%	11%	+/- 20.0%
Prednisone	77%	62%	93%	+/- 20.0%
**Dosage**				
	**Current value**	**Low value**	**High value**	**Source**
**Avatrombopag**				
Week 0–3	20.00	16.22	23.78	
Week 4–7	20.00	16.22	23.78	
Week 8–11	20.00	16.22	23.78	
Week 12–15	20.00	16.22	23.78	
Week 16–19	20.00	16.22	23.78	Avatrombopag Phase 3 trial [9]
Week 20–23	20.00	16.22	23.78	
Week 24–27	20.00	16.22	23.78	

Post-week 28	20.00	16.22	23.78	
Frequency per 4-week cycle	28.00	22.40	33.60	+/- 20.0%
**Eltrombopag**				
Week 0–3	50.00	45.00	55.00	+/- 10.0%
Week 4–7	50.00	45.00	55.00	+/- 10.0%
Week 8–11	50.00	45.00	55.00	+/- 10.0%
Week 12–15	50.00	45.00	55.00	+/- 10.0%
Week 16–19	50.00	45.00	55.00	+/- 10.0%
Week 20–23	50.00	45.00	55.00	+/- 10.0%
Week 24–27	50.00	45.00	55.00	+/- 10.0%
Post-week 28	50.00	45.00	55.00	+/- 10.0%
Frequency per 4-week cycle	28.00	22.40	33.60	+/- 20.0%
**Romiplostim**				
Week 0–3	0.004	0.003671507	0.004328493	
Week 4–7	0.004	0.003671507	0.004328493	
Week 8–11	0.004	0.003671507	0.004328493	
Week 12–15	0.004	0.003671507	0.004328493	Kuter et al. 2008 [21]
Week 16–19	0.004	0.003671507	0.004328493	
Week 20–23	0.004	0.003671507	0.004328493	
Week 24–27	0.004	0.003671507	0.004328493	
Post-week 28	0.004	0.003671507	0.004328493	
Frequency per 4-week cycle	4	3.2	4.8	+/- 20.0%
	**Current value**	**Low value**	**High value**	**Source**
**% of patients with bleeding per cycle, by type and response**				
Platelets ≥50 × 10^9/L – Minor bleed	10.0%	8.0%	12.0%	+/- 20.0%
Platelets ≥50 × 10^9/L – Outpatient bleed	7.1%	5.7%	8.5%	+/- 20.0%
Platelets ≥50 × 10^9/L – Inpatient bleed	0.0%	0.0%	0.0%	+/- 20.0%
Platelets <50 × 10^9/L – Minor bleed	17.1%	13.7%	20.6%	+/- 20.0%
Platelets <50 × 10^9/L – Outpatient bleed	45.5%	36.4%	54.6%	+/- 20.0%
Platelets <50 × 10^9/L – Inpatient bleed	4.3%	3.4%	5.2%	+/- 20.0%
**Time to response (cycles)**				
Avatrombopag	6.00	5.00	7.00	+/- 1
Eltrombopag	6.00	5.00	7.00	+/- 1
Romiplostim	6.00	5.00	7.00	+/- 1
**Response rate**				
Avatrombopag	73%	59%	88%	+/- 20.0%
Eltrombopag	27%	22%	33%	+/- 20.0%

Romiplostim	55%	44%	66%	+/- 20.0%
**Time on response (cycles)**				
Avatrombopag	109	87	130	+/- 20.0%
Eltrombopag	109	87	130	+/- 20.0%
Romiplostim	109	87	130	+/- 20.0%
**% of patients using rescue therapy**				
Platelets ≥50 × 10^9/L	3%	2%	4%	+/- 20.0%
Platelets <50 × 10^9/L	22%	18%	26%	+/- 20.0%
**Rescue therapy, Response rate**				
IVIg	80%	64%	96%	+/- 20.0%
IV steroids	46%	37%	55%	+/- 20.0%
Platelet transfusion	52%	42%	62%	+/- 20.0%
**% of patients using concomitant ITP medications in response and no response states**				
Among patients without response to active treatment	45%	36%	54%	+/- 20.0%
Without dose reduction	30%	24%	36%	+/- 20.0%
With dose reduction	6%	5%	7%	+/- 20.0%
Ratio of reduction in dose	5%	4%	6%	+/- 20.0%
**Probability of serious TRAEs**				
Avatrombopag	3%	2%	4%	+/- 20.0%
Eltrombopag	3%	2%	4%	+/- 20.0%
Romiplostim	3%	2%	4%	+/- 20.0%
Rescue – IVIg	2%	2%	2%	+/- 20.0%
Rescue – IV corticosteroid	3%	2%	4%	+/- 20.0%
Rescue – platelet transfusion	0.011%	0.009%	0.014%	+/- 20.0%
**Probability of other TRAEs**				
Avatrombopag	31%	25%	37%	+/- 20.0%
Eltrombopag	31%	25%	37%	+/- 20.0%
Romiplostim	31%	25%	37%	+/- 20.0%
Rescue – IVIg	0%	0%	0%	+/- 20.0%
Rescue – IV corticosteroid	70%	56%	84%	+/- 20.0%
Rescue – platelet transfusion	0%	0%	0%	+/- 20.0%
Costs				
	**Current value**	**Low value**	**High value**	**Source**
**Cost per pack**				
Active treatment – Avatrombopag, 30 × 20 mg	1920.00	1536.00	2304.00	+/- 20.0%
Active treatment – Eltrombopag, 28 × 50 mg	1540.00	1232.00	1848.00	+/- 20.0%

Active treatment – Romiplostim, 0.125 mg	241.00	192.80	289.20	+/- 20.0%
Rescue therapy – IVIg	50.00	40.00	60.00	+/- 20.0%
Rescue therapy – IV steroid	88.81	71.05	106.57	+/- 20.0%
Rescue therapy – platelet transfusion	0	0	0	
Concomitant ITP medications – Danazol	36.32	29.06	43.58	+/- 20.0%
Concomitant ITP medications – Azathioprine	2.57	2.06	3.08	+/- 20.0%
Concomitant ITP medications – Cyclosporine	18.37	14.70	22.04	+/- 20.0%
Concomitant ITP medications – Etamsylate	9.00	7.20	10.80	+/- 20.0%
Concomitant ITP medications – Dexamethasone	49.00	39.20	58.80	+/- 20.0%
Concomitant ITP medications – Prednisolone	2.41	1.93	2.89	+/- 20.0%
Concomitant ITP medications – Prednisone	2.41	1.93	2.89	+/- 20.0%
**Administration – 1 cycle**				
Active treatment – Avatrombopag, 30 × 20 mg	0	0	0	+/- 20.0%
Active treatment – Eltrombopag, 28 × 50 mg	0	0	0	+/- 20.0%
Active treatment – Romiplostim, 0.125 mg	45.86	36.69	55.04	+/- 20.0%
Rescue therapy – IVIg	241.06	192.85	289.27	+/- 20.0%
Rescue therapy – IV steroid	195.66	156.53	234.79	+/- 20.0%
Rescue therapy – platelet transfusion	889.86	711.73	1067.59	+/- 20.0%
Concomitant ITP therapies	0	0	0	
**Administration subsequent cycle**				
Active treatment – Avatrombopag, 30 × 20 mg	0	0	0	+/- 20.0%
Active treatment – Eltrombopag, 28 × 50 mg	0	0	0	+/- 20.0%
Active treatment – Romiplostim, 0.125 mg	20.70	16.56	24.84	+/- 20.0%
Concomitant ITP medications	0	0	0	+/- 20.0%
**Cost of follow-up**				
Unit cost – Haematologist consultation	173.39	138.71	208.07	+/- 20.0%
Unit cost – Blood test	2.79	2.23	3.35	+/- 20.0%
Unit cost – Biochemistry	1.10	0.88	1.32	+/- 20.0%
Occurrence/month – Haematologist consultation	1.00	0.80	1.20	+/- 20.0%
Occurrence/month – Blood test	1.00	0.80	1.20	+/- 20.0%
Occurrence/month – Biochemistry	1.00	0.80	1.20	+/- 20.0%
Bleed cost				
Minor bleed	0	0	0	+/- 20.0%
Outpatient bleed	494.00	395.20	592.80	+/- 20.0%
Intracranial haemorrhage	7044.00	5635.20	8452.80	+/- 20.0%
Gastrointestinal	5503.00	4402.40	6603.60	+/- 20.0%
Other inpatient bleed	3485.00	2788.00	4182.00	+/- 20.0%

Utility				
	**Current value**	**Low value**	**High value**	**Source**
**Health-related quality of life**				
Platelets ≥50 × 10^9/L, No bleed	0.800793265	0.640634612	0.960951918	+/- 20.0%
Platelets ≥50 × 10^9/L, Minor bleed	0.755833265	0.604666612	0.906999918	+/- 20.0%
Platelets ≥50 × 10^9/L, Outpatient bleed	0.624993265	0.499994612	0.749991918	+/- 20.0%
Platelets ≥50 × 10^9/L, Intracranial haemorrhage	0.038	0.0304	0.0456	+/- 20.0%
Platelets ≥50 × 10^9/L, Gastrointestinal bleed	0.45	0.36	0.54	+/- 20.0%
Platelets ≥50 × 10^9/L, Other inpatient bleed	0.45	0.36	0.54	+/- 20.0%
Platelets <50 × 10^9/L, No bleed	0.760123265	0.608098612	0.912147918	+/- 20.0%
Platelets <50 × 10^9/L, Minor bleed	0.715163265	0.572130612	0.858195918	+/- 20.0%
Platelets <50 × 10^9/L, Outpatient bleed	0.584323265	0.467458612	0.701187918	+/- 20.0%
Platelets <50 × 10^9/L, Intracranial haemorrhage	0.038	0.0304	0.0456	+/- 20.0%
Platelets <50 × 10^9/L, Gastrointestinal bleed	0.45	0.36	0.54	+/- 20.0%
Platelets <50 × 10^9/L, Other inpatient bleed	0.45	0.36	0.54	+/- 20.0%
**Utility decrement with serious TRAEs**				
Avatrombopag	0.1	0.05	0.15	+/- 20.0%
Eltrombopag	0.1	0.05	0.15	+/- 20.0%
Romiplostim	0.1	0.05	0.15	+/- 20.0%
Rescue – IVIg	0.1	0.05	0.15	+/- 20.0%
Rescue – IV steroid	0.1	0.05	0.15	+/- 20.0%
Rescue – platelet transfusion	0.1	0.05	0.15	+/- 20.0%
**Utility decrement with other TRAEs**				
Avatrombopag	0.1	0.08	0.12	+/- 20.0%
Eltrombopag	0.1	0.08	0.12	+/- 20.0%
Romiplostim	0.1	0.08	0.12	+/- 20.0%
Rescue – IVIg	0.1	0.08	0.12	+/- 20.0%
Rescue – IV steroid	0.1	0.08	0.12	+/- 20.0%
Rescue – platelet transfusion	0.1	0.08	0.12	+/- 20.0%
**Mortality**				
	**Current value**	**Low value**	**High value**	**Source**
**Mortality of inpatient bleed**				
Intracranial haemorrhage	13%	1%	2%	
Gastrointestinal bleed	5%	3%	6%	Danese 2009 [22]
Other inpatient bleed	2%	10%	17%	

ITP: immune thrombocytopenia, IV: intravenous, IVIg: intravenous immunoglobulin, TRAE: treatment-related adverse event.

Table S7 Distribution of later-line treatments after non-response or loss of response to treatment I (avatrombopag, eltrombopag or romiplostim).

Treatment	Treatment II	Treatment III	Treatment IV
Avatrombopag	0%	0%	0%
Eltrombopag	0%	0%	0%
Romiplostim	0%	0%	0%
Rituximab	23%	16%	0%
Splenectomy	12%	13%	0%
Watch and wait	39%	53%	100%
Cyclosporine	4%	3%	0%
Danazol	4%	3%	0%
Cyclophosphamide	4%	3%	0%
Vincristine	4%	3%	0%
Vinblastine	4%	3%	0%

Table S8 Results of scenario analyses

(a) Avatrombopag versus eltrombopag

Scenario analysis	Total QALYs	Incremental QALYs	Total cost (£)	Incremental cost (£)	ICER (cost per QALY)
**OR for durable platelet response: Bayesian framework**					
Bleeding costs: standard NHS tariff[a]	Avatrombopag: 10.979<br>Eltrombopag: 10.085	0.894	Avatrombopag: 300,919<br>Eltrombopag: 292,643	8277	9259
Bleeding costs: qualitative study data[a]	Avatrombopag: 10.979<br>Eltrombopag: 10.085	0.894	Avatrombopag: 663,023<br>Eltrombopag: 705,187	−42,164	Dominant
Subsequent lines of treatment included[b]	Avatrombopag: 11.326<br>Eltrombopag: 10.491	0.836	Avatrombopag: 312,115<br>Eltrombopag: 306,042	6073	7268
Treatment response period of 12 weeks	Avatrombopag: 11.010<br>Eltrombopag: 10.101	0.908	Avatrombopag: 317,071<br>Eltrombopag: 310,326	6745	7424
**OR for durable platelet response: Frequentist framework**					
Bleeding costs: increased NHS tariff[a] (base-case)	Avatrombopag: 10.362<br>Eltrombopag: 9.976	0.385	Avatrombopag: 306,689<br>Eltrombopag: 309,195	–2506	Dominant
Bleeding costs: standard NHS tariff[a]	Avatrombopag: 10.362<br>Eltrombopag: 9.976	0.385	Avatrombopag: 286,255<br>Eltrombopag: 287,498	–1243	Dominant
Bleeding costs: qualitative study data[a]	Avatrombopag: 10.362<br>Eltrombopag: 9.976	0.385	Avatrombopag: 683,140<br>Eltrombopag: 706,123	–22,983	Dominant
Subsequent lines of treatment included[b]	Avatrombopag: 10.750<br>Eltrombopag: 10.389	0.360	Avatrombopag: 299,003<br>Eltrombopag: 301,197	–2194	Dominant
Treatment response period of 12 weeks	Avatrombopag: 10.383<br>Eltrombopag: 9.991	0.392	Avatrombopag: 304,223<br>Eltrombopag: 305,472	–1249	Dominant

(b) Avatrombopag versus romiplostim

Scenario analysis	Total QALYs	Incremental QALYs	Total cost (£)	Incremental cost (£)	ICER (cost per QALY)
**OR for durable platelet response: Bayesian approach**					
Bleeding costs: standard NHS tariff[a]	Avatrombopag: 10.979<br>Romiplostim: 10.628	0.351	Avatrombopag: 300,919<br>Romiplostim: 386,797	−85,877	Dominant
Bleeding costs: qualitative study data[a]	Avatrombopag: 10.979<br>Romiplostim: 10.628	0.351	Avatrombopag: 663,023<br>Romiplostim: 768,704	−105,681	Dominant
Subsequent lines of treatment included[b]	Avatrombopag: 11.326<br>Romiplostim: 10.998	0.328	Avatrombopag: 312,115<br>Romiplostim: 398,858	−86,742	Dominant
Treatment response period of 12 weeks	Avatrombopag:11.010<br>Romiplostim: 10.653	0.357	Avatrombopag: 317,071<br>Romiplostim: 399,719	−82,648	Dominant
**OR for durable platelet response: Frequentist approach**					
Bleeding costs: increased NHS tariff[a] (base-case)	Avatrombopag: 10.362<br>Romiplostim: 10.466	−0.104	Avatrombopag: 306,689<br>Romiplostim:390,094	−83,405	801,848
Bleeding costs: standard NHS tariff[a]	Avatrombopag: 10.362<br>Romiplostim: 10.466	−0.104	Avatrombopag: 286,255<br>Romiplostim: 370,000	−83,746	805,125
Bleeding costs: qualitative study data[a]	Avatrombopag: 10.362<br>Romiplostim: 10.466	−0.104	Avatrombopag: 683,140<br>Romiplostim: 761,016	−77,877	748,701
Subsequent lines of treatment included[b]	Avatrombopag: 10.750<br>Romiplostim: 10.847	−0.097	Avatrombopag: 299,003<br>Romiplostim: 382,492	−83,489	858,704
Treatment response period of 12 weeks	Avatrombopag: 10.383<br>Romiplostim: 10.488	−0.106	Avatrombopag: 304,223<br>Romiplostim: 383,220	−78,997	747,313

[a]See supplementary appendix for details of bleeding costs; [b]Patients who did not respond to TPO-RA treatment (or whose initial response was not maintained) could receive up to three further lines of active (non-TPO-RA) treatment.

ICER: incremental cost-effectiveness ratio, OR: odds ratio, QALY: quality-adjusted life-year, NHS: National Health Service, TPO-RA: thrombopoietin receptor agonist.

Figure S1. Tornado chart for avatrombopag versus eltrombopag (deterministic sensitivity analysis)[a]

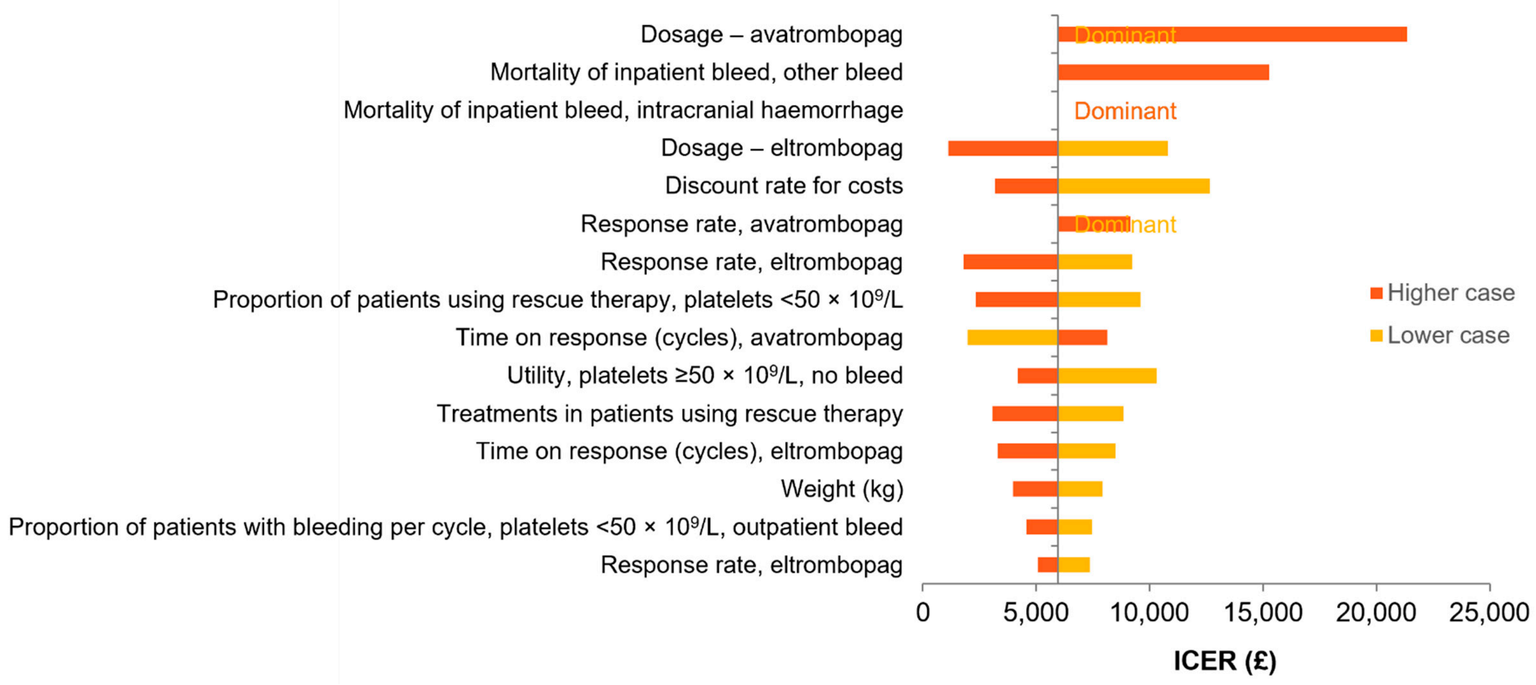

[a]Avatrombopag dominated romiplostim across all parameters varied in the deterministic sensitivity analysis, so Tornado plot not relevant.

ICER: incremental cost-effectiveness ratio.

References

1. National Health Service. NHS National Tariff Workbook 2019/2020 2020. Available online: https://www.england.nhs.uk/publication/past-national-tariffs-documents-and-policies/ (accessed on 30 June 2023).
2. Pogna E.A.; Middleton S.; Nazir J.; Ralph L.; Wilson K.; Jurczak W. Characterization and treatment of immune thrombocytopenia in Europe: a qualitative observational study. *Hematology*. **2021**, *26*, 860-869.
3. NHS England. 2019/20 National Cost Collection Data Publication 2020. Available online: https://www.england.nhs.uk/publication/2019-20-national-cost-collection-data-publication/ (accessed on 30 June 2023).
4. NHS Wales. Together for health – a delivery plan for the critically ill 2016. Available online: https://www.wales.nhs.uk/documents/Delivery-Plan-for-the-critically-ill.pdf (accessed on 30 June 2023).
5. University Hospitals Birmingham Trust. FOI 0778 Tariff/cost of bed 2018. Available online: https://hgs.uhb.nhs.uk/foi-0778-tariff-cost-of-bed/ (accessed on 30 June 2023).
6. London Ambulance Service NHS Trust. Annual report & accounts 2018/2019. Available online: https://www.londonambulance.nhs.uk/wp-content/uploads/2019/08/London-Ambulance-Service-Annual-Report-Accounts-2018-19.pdf (accessed on 30 June 2023).
7. National Institute for Health and Care Excellence (National Clinical Guideline Centre). Preoperative tests clinical guideline 2015. Available online: https://www.nice.org.uk/guidance/NG45/documents/guideline-appendices-13 (accessed on 30 June 2023).
8. National Institute for Health and Care Excellence. British National Formulary 2021. Available online: https://bnf.nice.org.uk (accessed on 30 December 2023).
9. Jurczak W.; Chojnowski K.; Mayer J.; Krawczyk K.; Jamieson B.D.; Tian W.; Allen L.F. Phase 3 randomised study of avatrombopag, a novel thrombopoietin receptor agonist for the treatment of chronic immune thrombocytopenia. *Br. J. Haematol*. **2018**, *183*, 479-490.
10. Sobi. Clinical study report: a phase 3, multicenter, randomized, double-blind, placebo-controlled, parallel-group trial with an open-label extension phase to evaluate the efficacy and safety of oral E5501 plus standard care for the treatment of thrombocytopenia in adults with chronic immune thrombocytopenia (idiopathic thrombocytopenic purpura). 2015.
11. National Institute for Health and Care Excellence. Romiplostim for the treatment of chronic immune thrombocytopenia. Technology appraisal guidance [TA221] 2018. Available online: https://www.nice.org.uk/guidance/ta221 (accessed on 30 April 2023).
12. National Institute for Health and Care Excellence. Eltrombopag for adult patients with chronic immune thrombocytopenic purpura (cITP). Single technology appraisal (STA) 2012. Available online: https://www.nice.org.uk/guidance/ta293/documents/thrombocytopenic-purpura-eltrombopag-rev-ta205-glaxosmithkline4 (accessed on 12 December 2023).
13. National Health Service. NHS National Tariff Workbook 2018/2019 2019. Available online: https://www.england.nhs.uk/publication/past-national-tariffs-documents-and-policies/ (accessed on 30 June 2023).
14. National Institute for Health and Care Excellence. Romiplostim for the treatment of chronic immune or idiopathic thrombocytopenic purpura (ITP): A Single Technology Appraisal 2008. Available online: https://www.nice.org.uk/guidance/ta221/documents/romiplostim-for-the-treatment-of-chronic-immune-or-idiopathic-thrombocytopenic-purpura-itp-a-single-technology-appraisal2 (accessed on 30 April 2023).
15. National Institute for Health and Care Excellence. Single technology appraisal. Avatrombopag in combination for treating chronic immune thrombocytopenia [ID3838] 2022. Available online: https://www.nice.org.uk/guidance/ta853/documents/committee-papers (accessed on 31 May 2023).
16. Provan D.; Stasi R.; Newland A.C.; Blanchette V.S.; Bolton-Maggs P.; Bussel J.B.; Chong B.H.; Cines D.B.; Gernsheimer T.B.; Godeau B. et al. International consensus report on the investigation and management of primary immune thrombocytopenia. *Blood*. **2010**, *115*, 168-186.
17. JoDrugs. Ethamsylate dosing 2023. Available online: http://www.jodrugs.com/products/38706-ethamsylate.aspx (accessed on 12 December 2023).
18. Liu Y.; Zhang H.X.; Su J.; Geng Q.C.; Lin X.; Feng C.X. Efficacy and incidence of treatment related adverse events of thrombopoietin receptor agonists in adults with immune thrombocytopenia: a

systematic review and network meta-analysis of randomized controlled study. *Acta. Haematol*. **2023**, *146*, 173-184.
19. Wojciechowski P.; Wilson K.; Nazir J.; Pustułka I.; Tytuła A.; Smela B.; Pochopien M.; Vredenburg M.; McCrae K.R.; Jurczak W. Efficacy and safety of avatrombopag in patients with chronic immune thrombocytopenia: a systematic literature review and network meta-analysis. *Adv. Ther*. **2021**, *38*, 3113-3128.
20. National Institute for Health and Care Excellence. NICE health technology evaluations: the manual 2022. Available online: https://www.nice.org.uk/process/pmg36/resources/nice-health-technology-evaluations-the-manual-pdf-72286779244741 (accessed on 31 October 2023).
21. Kuter D.J.; Bussel J.B.; Lyons R.M.; Pullarkat V.; Gernsheimer T.B.; Senecal F.M.; Aledort L.M.; George J.N.; Kessler C.M.; Sanz M.A. et al. Efficacy of romiplostim in patients with chronic immune thrombocytopenic purpura: a double-blind randomised controlled trial. *Lancet*. **2008**, *371*, 395-403.
22. Danese M.D.; Lindquist K.; Gleeson M.; Deuson R.; Mikhael J. Cost and mortality associated with hospitalizations in patients with immune thrombocytopenic purpura. *Am. J. Hematol*. **2009**, *84*, 631-635.
